# Merkel-Cell Carcinoma: Local Recurrence Rate Versus Radiation Dose Study from a 949-Patient Database †

**DOI:** 10.3390/curroncol32040202

**Published:** 2025-03-28

**Authors:** Patricia Tai, Michael Veness, Vimal H. Prajapati, Aoife Jones Thachuthara, Jidong Lian, Avi Assouline, Edward Yu, Kurian Joseph

**Affiliations:** 1 Department of Oncology, University of Saskatchewan, Saskatoon, SK S7N 5A2, Canada; 2 Department of Oncology, University of Sydney, Sydney, NSW 2050, Australia; michael.veness@health.nsw.gov.au; 3 Department of Dermatology, University of Calgary, Calgary, AB T2N 1N4, Canada; vimal.h.prajapati@dermatologyphilosophy.com; 4 Department of Medical Oncology, Cork University Hospital, T12 DC4A Cork, Ireland; aoife.jones3@hse.ie; 5 Department of Oncology, University of Toronto, Toronto, ON M5S 1A1, Canada; jidong.lian@thp.ca; 6 Department of Radiation Oncology, Centre de Cancérologie de la Porte de Saint-Cloud (CCPSC), 92100 Boulogne-Billancourt, France; avi.assouline@ccpsc.fr; 7 Department of Oncology, Western University, London, ON N6A 3K7, Canada; eyu@uwo.ca; 8 Department of Oncology, University of Alberta, Edmonton, AB T6G 2R3, Canada; kurian.joseph@ahs.ca

**Keywords:** Merkel-cell carcinoma, local recurrence, radiotherapy dose, pattern of spread, skin, database

## Abstract

(1) Background: Knowledge regarding the optimal radiotherapy dose for Merkel-cell carcinoma (MCC) remains limited. (2) Methods: Following a PubMed search, equivalent doses in 2 Gy fractions (Gy2) were compared. (3) Results: Of the 949 patients, 939 were evaluable, with 728 (77.5%) cases localized to the primary site and 171 irradiated without chemotherapy. The overall local recurrence rate (LRR) was 23% (40/171). After definitive radiotherapy with EQD2 < 50 Gy2 versus ≥50 Gy2, the LRRs were 23.1% (3/13) and 12.5% a(1/8), respectively (*p* = 0.0004). (4) Conclusions: For definitive radiotherapy, EQD2 < 50 Gy2 demonstrates a significantly higher LRR than ≥50 Gy2 (*p* = 0.0004). This study is clinically useful and unique with stratification by definitive/adjuvant settings and positive/negative resection margins. A future prospective multicenter study is needed to determine the optimal radiotherapy doses.

## 1. Introduction

Merkel-cell carcinoma (MCC) is an aggressive radiosensitive cutaneous neuroendocrine malignancy arising predominantly in older (70+-year-old) Caucasians. Many (30–40%) will die from MCC despite treatment, usually from distant metastases. The incidence is increasing rapidly, and lesions can arise anywhere on the body, with the head and neck involved in 40–50% of cases [1].

The etiology of MCC is associated with a highly prevalent virus, the Merkel-cell polyomavirus, as well as chronic ultraviolet radiation. The increasing global incidence is notable and may be attributed to greater awareness among clinicians [2]. Clinical diagnosis of MCC is difficult, although the AEIOU features may help [3]. Basal-cell carcinoma is the most common skin cancer and, generally, it is quite treatable. Melanoma is a well-known, lethal skin cancer if presented late, but the less-known MCC is even more lethal, with an average mortality of 30% when all stages are combined [4,5]. According to the 2013 data from the American National Cancer Database (NCDB), the five-year overall survival for clinical stage I is only 45.0% and approximately 30% for stage II [6]. Pathological stages have better results since more aggressive cases are moved to higher stages on pathological examination, and the outcomes are 62%, 40%, 33%, and 13.5%, respectively, from pathological stage I to IV [7]. Patients who present with nodal metastases with an unknown or occult primary tend to have a better outcome [8].

The differential diagnoses for small-round-blue-cell tumors are broad. Immunohistochemical markers (CK20+, CK7−, and TTF1−) are used to distinguish between MCC and other tumors. It is particularly important to distinguish MCC from its greatest mimic, small-cell carcinoma of the lung, as the latter can give rise to skin metastases [9]. Other differential diagnoses are skin T-cell lymphoma, Ewing’s sarcoma in adults, or even benign lesions like pyogenic granuloma [10,11]. It should be noted that mixed tumors can occur as so-called “collision” skin cancers [12,13]. MCC stains positive for common neuroendocrine markers, such as chromogranin A (CGA, the most specific marker), synaptophysin (SYP, the most sensitive marker), and neuron-specific enolase (NSE) [14].

The natural history of MCC influences our treatment approach. It has a tendency for rapid local progression and frequent spread to regional lymph nodes and distant sites. Due to the rarity of the disease, the optimal treatment has not been fully defined. Localized MCC (stages I and II) is treated by surgical excision of the primary tumor (with a 1 to 3 cm margin) and lymphadenectomy in cases of node-positive disease, followed by external beam radiotherapy to the tumor bed. Adjuvant radiotherapy has been shown to markedly decrease locoregional recurrence rates (LRRs) and to increase survival in recent studies [15,16].

The treatment of the regional nodal area is more controversial. MCC has a high propensity to spread to draining lymph nodes. It is relevant to establish the extent of a patient’s MCC at diagnosis as this will impact both management and prognosis. Most (50–60%) patients present with a primary lesion only but there is a need to investigate draining lymph nodes (LNs), so-called sentinel lymph nodes (SLNs), as the incidence of subclinical spread is high (30–50%). Around 20–30% of patients will present with nodal metastases [15]. Traditionally, node-positive patients have been treated with lymph nodal dissection (LND). However, radiotherapy can also serve as either a definitive or an adjuvant treatment option.

Being a cutaneous neuroendocrine tumor, MCC has been approached by clinicians with treatment strategies similar to those used for other neuroendocrine carcinomas, such as chemotherapy and radiotherapy. The management approach was ‘adopted’ based on experience of the treatment of small-cell lung cancer (SCLC), a more common neuroendocrine tumor. Different dose fractionations were used in the past for SCLC, from 40 Gy/15 fractions in 3 weeks to 70 Gy/35 fractions in 7 weeks [17]. The twice-a-day (BID) regimen of 45 Gy/30 fractions over 3 weeks was quite toxic [18]. In a 2024 review of the literature, a scatter plot of biologic equivalent doses (BED10) versus two-year overall survival rates showed a *p*-value of 0.0001, indicating that survival improved with higher doses. This supports the principle of biologic plausibility, as small-cell lung cancer tends to recur after treatment. A higher dose may result in a more profound or “deeper” complete response, which is a common concept in systemic treatment. Consequently, a higher dose may also lead to lower local recurrence.

There has, to date, been limited research on the optimal radiotherapy doses for microscopic and macroscopic diseases such as MCC. Hence, researchers have given different recommendations [19]. In 1990, based on a 54-patient study from the M.D. Anderson Cancer Center, the authors recommended a dose of 56–60 Gy for gross disease and 46–50 Gy for adjuvant radiotherapy, both in 2 Gy fractions [20]. A 2008 review recommended an adjuvant dose of 50–60 Gy for the tumor bed [21].

Two analyses of the NCDB reported different results for adjuvant radiotherapy doses. One study, published in 2017, concluded that doses ranging from 40 to <50 Gy appear to be sufficient for extremities and/or the trunk in stage I to III MCC, with overall survival equivalent to that of higher-dose regimens (>50–70 Gy). [22]. However, another study, published in 2020, supported doses of 50–57 Gy for most stage I/II Merkel-cell carcinoma patients receiving adjuvant radiotherapy, assuming 2 Gy per fraction is used [23]. Its authors strongly recommended ≥50 Gy for patients with nodal presentations irrespective of the tumor site. Therefore, it appears that there are two schools of thought for adjuvant treatment—the lower 40 to <50 Gy and the higher 50–60 Gy. MCC is a rare cancer and so randomized studies are difficult to organize [24]. One notable prospective study was conducted by Jouary et al. [25]. To strike a balance between the options, the experts in the updated German S2k guideline of 2023 stated that the standard dose for adjuvant radiotherapy remains 50 Gy [26].

When combined with chemotherapy, the radiotherapy dose employed can be lowered. The Trans-Tasman Radiation Oncology Group study combined chemotherapy with 50 Gy in 25 fractions over 5 weeks to *gross* primaries and nodes [27]. Nodal involvement was present in 14/18, i.e., 77% of patients. Eligibility criteria included at least one of the following high-risk features: recurrence after initial therapy, involved nodes, primary size greater than 1 cm, gross residual disease after surgery, or occult primary with nodes [28]. However, combination chemo-radiation has recently fallen out of favor due to its toxicities and the theoretical concern that chemotherapy-induced immunosuppression may adversely impact prognosis. Currently, research on immunotherapy in MCC represents the leading trend.

The next issue is the effect of dose fractionation. The group at Dana-Farber/Brigham and Women’s Cancer Center studied 241 patients with non-metastatic MCC, treated with conventional (conv-RT) or hypo-fractionated radiotherapy (hypo-RT) [29]. The hypo-RT cohort was older (≥73 years at diagnosis: 78.0% vs. 41.5%, *p* < 0.01) and received a lower equivalent total RT dose in 2 Gy per fraction (<50 Gy: 58.0% vs. 5.2%, *p* < 0.01). Median follow-up was 65.1 months (range: 1.2–194.5 months) for conv-RT and 25.0 months (range: 1.6–131.3) for hypo-RT cohorts. The two-year cumulative incidence of in-field locoregional relapse was low in both groups (1.1% conv-RT vs. 4.1% hypo-RT, *p* = 0.11). While the two-year overall survival was lower for the hypo-RT group (62.6% vs. 84.4%, *p* = 0.0008), the two-year MCC-specific survival was similar (84.7% vs. 86.6%, *p* = 0.743).

With the above-mentioned controversies, our research team decided to examine the optimal dose fractionation for MCC using the EQD2 concept, i.e., the equivalent dose if converted to 2 Gy fractions (Gy2) based on the linear quadratic formula [30]. This EQD2 concept helps us compare different dose fractionations used in various centers against LRR, filling the gap in knowledge for MCC.

## 2. Materials and Methods

We built a 949-patient aggregated database from the records of our institutions and individual patient data from the literature for the period March 1982 to Feb 2015. This includes a retrospective chart review of data from six jurisdictions across France, Canada, and Australia. A PubMed search was also conducted to ensure an adequate database. A Microsoft Excel datasheet was designed. There were no exclusion criteria for patients; however, during the dose-fractionation analysis, cases with insufficient details were not included in the computations.

The following were compiled for patients after ethics approval—baseline information of age, sex, initial clinical and pathological stages, site, time delay before seeing doctors, other concurrent tumor(s), maximum dimension of the primary tumor, nodal and distant metastases, histological details, and a history of immunosuppression/co-morbidities/previous radiotherapy. We recorded the treatment(s) received—surgery (e.g., Mohs microsurgery, nodal dissection or excision alone, and resection margin), radiotherapy (doses, field coverage, and response), chemotherapy (specific chemotherapy drugs, number of cycles, and response), and the outcome such as recurrence (timing, site, and subsequent treatments), and final disease status. MCC polyomavirus status was not included due to non-availability at the time of the study.

The primary outcome was lifetime local recurrence rates (LRR). Equivalent doses in 2 Gy fractions (EQD2) = total dose × [(dose per fraction + α/β)/(2 + α/β)] were calculated to compare different dose fractionations, assuming α/β = 10 [11].

### Statistical Analysis

Lifetime LRRs were analyzed as the primary outcome, with comparison by the unpaired t-test, chi-square, and Fisher exact tests [31,32,33]. The secondary outcomes were survival rates. Cause-specific survival was defined as the time interval from diagnosis to death from MCC, or censored at the last follow-up date if the patient was still alive at the time of analysis. Overall survival was defined as the time interval from diagnosis to death regardless of the cause, or last follow-up date for censoring as described above. The Kaplan–Meier method was used to generate survival curves [34]. The Cox proportional hazards model was used to identify risk factors [35].

## 3. Results

Altogether, 939 out of 949 data points were evaluable, with sufficient radiotherapy information for calculations—50.8% were male, the median age was 73 years (range: 31–96), and the median follow-up was 21 months (range: 0–272). Of the 939 patients, 728 (77.5%) presented with localized disease (stages I and II), and 176 (18.7%) with nodal disease (stage III). A median dose of 50 Gy2 (range: 14–70) was used for both micro- and macroscopic tumors. Among the 171 stage I and II patients who were irradiated without chemotherapy, median primary EQD2 was 50 (14.0–72.0) Gy2, and nodal EQD2 was 50 (15.9–71.9) Gy2. The five-year Kaplan–Meier cause-specific survival was 56.5%; overall survival was 43.8%; and LRR was 23.4% (40/171) after a median EQD2 of 50 (14.0–70.0) Gy2. The remaining patients (out of the total number of patients, excluding those who experienced local recurrence) achieved local control with a median EQD2 of 50 Gy2 (range: 23.3–72.0).

Table 1 shows that among patients receiving definitive radiotherapy with known doses for gross primary disease: LRR for EQD2 < 50 Gy2 vs. ≥50 Gy2 were 23.1% (3/13) vs. 12.5% (1/8) (*p* = 0.0004, student-*t* test). The comparison for <60 Gy2 vs. ≥60 Gy2 was not performed due to the small number of patients receiving ≥60 Gy2. Our complete response rate was 91.4% (32 out of 35 with documented response after radiotherapy to gross disease).

Adjuvant radiotherapy was given to 156 patients to the primary site, the LRRs with 50 Gy2 as the cut-off were 18.8% (6/32) vs. 12.8% (12/124) (*p* = 0.15 with both Fisher exact test and chi-square test); and for the 60 Gy2 cut-off, 15.5% (16/103) vs. 8.7% (2/23) (*p* = 0.40, chi-square test).

In patients with positive margins, the LRR was 25% (3/12) in <50 Gy2 and 15% (3/20) in ≥50 Gy2 group (*p* = 0.65, Fisher’s exact test). In patients with negative margins, the LRR was 17.4% (4/23) in <50 Gy2 and 4.8% (3/62) in ≥50 Gy2 group (*p* = 0.08, Fisher’s exact test).

Table 2 on stage III (node-positive) patients is provided here for the sake of completeness and further details will be the subject of another future publication.

## 4. Discussion

To our knowledge, this MCC database is one of the most comprehensive in the literature, providing extensive treatment details based on real-world experience. Radiotherapy doses for MCC should be individualized to achieve the best outcomes, taking into account specific body sites. For example, the shin tolerates radiation poorly due to its scant subcutaneous tissue, its location being prone to trauma, and its poor circulation. The cosmetic outcome is another important consideration for the face and hands. An excellent functional outcome is important for hands and feet. A relevant literature review for radiotherapy of MCC is presented below.

### 4.1. Sentinel Lymph Node Biopsy (SLNB) and Adjuvant Radiotherapy

Conservative surgery can be performed for both primary and nodal MCC. SLNB can be seen as a form of selective lymphadenectomy of involved nodes which is followed by radiation, being used in most American centers; for example, the University of Washington [36].

### 4.2. Effectiveness of Radiotherapy for Gross Disease

MCC has an excellent response to radiotherapy highlighting its unique radio-responsiveness among skin cancers. In a series from the Peter MacCallum Cancer Institute, Australia, complete responses of measurable tumors were observed in 22 out of 23 sites (96%) and 1 partial response (4%), i.e., an overall response rate of 100%. There was only 1 recurrence in an irradiated site (after a low radiation dose) [37]. More examples of success stories were found in the older literature [38], including a 26 cm tumor with excellent response [39]. Other studies have shown similarly excellent results on longer follow-up—75% at 49 months [40] and 85% at 2 years [41]. Modern radiotherapy techniques with stereotactic radiosurgery allow a higher dose to be achieved for the gross tumor while sparing surrounding normal tissues. This can be performed for liver, bone, and brain metastases < 3 cm in general [42].

In 2015, the Peter MacCallum Cancer Centre, Melbourne, Australia, published its 15 years of experience of positron emission tomography (PET) scans [43] and reported post-treatment metabolic response in 1–6 months to be significantly associated with improved overall survival. The 24/37 patients achieving a complete metabolic response had an 88% 2-year overall survival (95% CI, 0.75–1.00) and a 68% 5-y overall survival (95% CI, 0.49–0.95). Patients without a complete metabolic response had a 15% 1-year overall survival (95% CI, 0.04–0.55). PET scans are also useful for identifying locoregional recurrence which can be salvaged by multi-modality treatment [44,45]. The authors reported that four out of nine patients with locoregional recurrence were successfully salvaged through further treatment, with a median follow-up duration of 6.5 years.

### 4.3. Dose Response to Radiotherapy

To date, the optimum radiation doses for MCC are only estimated in the adjuvant, microscopic, and macroscopic settings. There are limited radiotherapy dose response data but an Australian study documented a dose response for patients with macroscopic disease and recommended 55 Gy as a minimum dose [45]. In that study, no patient with macroscopic MCC disease developed in-field relapse at doses >56 Gy [46]. The majority of patients treated with definitive RT had an out-of-field relapse with in-field control.

A systematic review of the literature reported an almost 90% in-field control rate following definitive radiotherapy with a mean delivered dose of just under 50 Gy, with no apparent association between RT dose and incidence of recurrence [47], likely due to the radiosensitivity of MCC. In an analysis of 2093 patients from the NCDB with MCC located on the trunk or extremities, all patients underwent surgery (88% achieved negative margins and 12% had microscopic or gross residual MCC) followed by adjuvant RT. Using overall survival as the outcome, patients receiving between 40 and 50 Gy had an equivalent survival to those receiving >50–70 Gy. Patients receiving <40 Gy had a worse survival [22].

Patients with a poor performance status should be considered for shorter hypo-fractionated regimes such as 20–30 Gy in 5–10 fractions. At least one study has documented a 45% complete response using an 8 Gy single fraction (including large tumors up to 16 cm) and almost 80% in-field lesion control [48]. Whether larger doses per fraction compensate for a lower total dose remains unclear; however, this approach serves as an alternative for selected patients with a poor performance status.

With combination treatment, chemotherapy appears to be effective when paired with 50 Gy of radiation [27,28]. A phase II trial randomized 50 patients to nivolumab 240 mg intravenously every 2 weeks plus ipilimumab 1 mg/kg intravenously every 6 weeks (group A) or the same drug schedule with the addition of stereotactic radiotherapy to at least one tumor site (24 Gy in three fractions at week 2; group B) to the gross tumor [49]. A total of 22 patients naïve to immune checkpoint inhibitors all showed a response to combined nivolumab and ipilimumab, of which 9 (41%) were complete responses. The authors concluded that the addition of stereotactic radiotherapy did not improve the efficacy of combined nivolumab and ipilimumab. However, we felt that the radiation dose used—36 Gy2—may have been insufficient, and a higher dose could potentially lead to a different conclusion. More research on combination immunotherapy and radiotherapy is needed to clarify the optimal dose.

Deducing from all this information (the present study and the literature) [50] and the arguments stated in the next section, clinicians may consider a higher dose for lesions with a greater concern for recurrence, e.g., higher stage, immunosuppression, truncal location, a delay in commencing treatment after diagnosis [51,52], a polyomavirus-negative tumor [53], multiple positive resection margins, recurrent lesions [27,28], multiple recurrent episodes, and patients who may not attend future follow-up [27,28,29,30,51,52,53,54,55,56]. All treatment decisions should be individualized to achieve the best outcomes. This study aims to assist clinicians who have limited experience with this rare tumor.

### 4.4. Implications of Present Study

As mentioned in the introduction, there appear to be two schools of thought for adjuvant treatment—the lower 40 to <50 Gy and the higher 50–60 Gy. MCC is a rare cancer and so randomized studies are difficult to organize. Overall, the authors of the present study favor the higher doses of 50–60 Gy. The LRR is reduced by half when using >60 Gy2 (8.7%) compared to <60 Gy2 (15.5%). The LRR of ≥60 Gy2 (8.7%) is lower than that of ≥50 Gy2 (12.8%). For all comparisons, lower LRRs were consistently observed with higher doses, even in adjuvant radiotherapy for microscopic disease. It is biologically plausible that gross disease may benefit from higher doses due to the larger tumor bulk. Future large prospective studies are required to determine the optimal doses, although such studies are unlikely to be conducted. Our conclusions may serve as suggestions and be hypothesis-generating rather than definitive evidence.

The present study corroborates the existing literature. The complete response rate was 91.4% (32/35) for gross disease, which compares favorably with the 96% reported by the Peter MacCallum Cancer Institute in Australia [37].

### 4.5. Future Research

Future research with large, prospective multicenter studies is needed to determine optimal radiotherapy doses and to evaluate the roles [57] of radiosensitizers [57], immunotherapy [58], and liquid biopsy [54]. Sharing experience among experts is also important to advance care for such rare cancers [55,56].

## 5. Conclusions

In summary, this study suggests that standard fractionated radiotherapy doses greater than 50 Gy, and possibly exceeding 60 Gy, may improve locoregional control. However, these findings should be viewed as suggestions and hypothesis-generating rather than definitive evidence. All treatment decisions should be individualized to achieve the best outcome. This study is meant to help clinicians who have little experience with this rare tumor. Future research with large, prospective multicenter studies is needed to determine optimal radiotherapy doses and to evaluate the roles of radiosensitizers, immunotherapy, and liquid biopsy in MCC.

## Figures and Tables

**Table 1:** Radiation doses after radiotherapy without chemotherapy for clinical stage I/II Merkel-cell carcinoma (primary disease only without nodes at presentation).

Presentation with Skin Primary Disease Only	Patient No. Median Definitive Dose (Range) Gy2	Patient No. with Known Margin Status. Median Adjuvant Dose (Range) Gy2	
Radiotherapy to primary site	N = 23 48.8 (31.3–60.0)	Margin −ve N = 81 55.6 (32.5–56.4)	Margin +ve N = 23 48.8 (31.3–60.0)

Abbreviations: −ve, negative; +ve, positive; Gy2, equivalent doses in 2 Gy fractions; no. number.

**Table 2:** Radiotherapy without chemotherapy for clinical stage III Merkel-cell carcinoma.

Presentation with Nodal Disease	Patient No. Median Definitive Dose (Range) Gy2	Patient Number with Known Margin Status Median Adjuvant Dose (Range) Gy2	
Radiotherapy to primary site	N = 20 58.5 (23.3–60.0)	Margin −ve N = 33 52.0 (50.0–66.7)	Margin +ve N = 15 50.0 (50.0–50.0)

Abbreviations: −ve, negative; +ve, positive; Gy2, equivalent doses in 2-Gy fractions; no. number.

## Data Availability

Data are unavailable to the public due to privacy (restrictions by the ethics committee above).

## Abbreviations

The following abbreviations are used in this manuscript:

ADMEC-O	Adjuvant immunotherapy with nivolumab versus observation
ADT	Androgen deprivation therapy
CSS	Cause-specific survival
CT	Computerized tomography
DFS	Disease-free survival
DM	Distant metastases
Gy2	Equivalent doses in 2 Gy fractions
LNM	Lymph node metastases
MCC	Merkel-cell carcinoma
OS	Overall survival
PET	Positron emission tomography
PFS	Progression-free survival
SLNB	Sentinel lymph node biopsy
−ve	Negative
+ve	Positive
